# Piscivore-Prey Fish Interactions: Mechanisms behind Diurnal Patterns in Prey Selectivity in Brown and Clear Water

**DOI:** 10.1371/journal.pone.0102002

**Published:** 2014-11-07

**Authors:** Lynn Ranåker, Jens Persson, Mikael Jönsson, P. Anders Nilsson, Christer Brönmark

**Affiliations:** 1 Department of Biology, Aquatic Ecology, Ecology Building, Lund University, Lund, Sweden; 2 Swedish Agency for Marin and Water Management, Gothenburg, Sweden; 3 Department of Biology, Functional zoology, Biology Building, Lund University, Lund, Sweden; 4 Department of Environmental and Life Sciences, Biology, Karlstad University, Karlstad, Sweden; Lund University, Sweden

## Abstract

Environmental change may affect predator-prey interactions in lakes through deterioration of visual conditions affecting foraging success of visually oriented predators. Environmental change in lakes includes an increase in humic matter causing browner water and reduced visibility, affecting the behavioural performance of both piscivores and prey. We studied diurnal patterns of prey selection in piscivorous pikeperch (*Sander lucioperca*) in both field and laboratory investigations. In the field we estimated prey selectivity and prey availability during day and night in a clear and a brown water lake. Further, prey selectivity during day and night conditions was studied in the laboratory where we manipulated optical conditions (humic matter content) of the water. Here, we also studied the behaviours of piscivores and prey, focusing on foraging-cycle stages such as number of interests and attacks by the pikeperch as well as the escape distance of the prey fish species. Analyses of gut contents from the field study showed that pikeperch selected perch (*Perca fluviatilis*) over roach (*Rutilus rutilus*) prey in both lakes during the day, but changed selectivity towards roach in both lakes at night. These results were corroborated in the selectivity experiments along a brown-water gradient in day and night light conditions. However, a change in selectivity from perch to roach was observed when the optical condition was heavily degraded, from either brown-stained water or light intensity. At longer visual ranges, roach initiated escape at distances greater than pikeperch attack distances, whereas perch stayed inactive making pikeperch approach and attack at the closest range possible. Roach anti-predatory behaviour decreased in deteriorated visual conditions, altering selectivity patterns. Our results highlight the importance of investigating both predator and prey responses to visibility conditions in order to understand the effects of degrading optical conditions on piscivore-prey interaction strength and thereby ecosystem responses to brownification of waters.

## Introduction

Predation by piscivorous fish is an important structuring force in freshwater food webs, where piscivory can cause complex trophic cascades with repercussions at the community and ecosystem levels [1]–[5]. Piscivore foraging can be divided into different foraging-cycle stages, including encounter, reaction, attack, capture and ingestion of prey [6], [7]. Theoretical foraging models suggest that encounter rate is a function of search volume and prey density [8]–[10], where search volume, in turn, is a function of reaction distance and swimming speed. For visually hunting piscivores, reaction distance is affected by environmental factors such as ambient light levels and turbidity as well as characteristics of both predator and prey species. Several studies of piscivores have shown that reaction distance decreases with increasing turbidity [8], [9], [11]–[13]. Further, optical conditions of the water may also affect attack rate [14], [15] and prey selection [11], [16]. From the prey’s perspective, deteriorated optical properties can induce reduced escape success due to poor timing of escape responses [17], but turbid water may also act as a refuge from visual predators [10]. The optical qualities of water hence hold an important key to our understanding of piscivore-prey fish interactions.

Most studies on how optical conditions affect piscivore foraging success focus on the effects of light intensity or turbidity caused by increasing levels of clay particles or algae [18], [19]. However, in recent years it has been recognised that increasing inputs of humic matter makes our lakes browner [20]–[22]. The loading of humic substances from terrestrial into aquatic systems is expected to increase in the future due to changes in land use, climate change [22] and reduced sulphur deposition [21], [23]. This brownification [20] of our inland as well as coastal waters may have far-reaching effects on biotic interactions and ecosystem functions [24], [25]. Humic substances have strong effects on the light climate in the water column by attenuating light, mainly in the UV and blue/green region, resulting in a light spectrum that is dramatically different from non-humic waters [26], [27]. The reduced light climate will cause negative effect on the contrasts between objects and their background, resulting in a reduced reaction distance of both predator and prey detection [28]. The effect of brownification on piscivore-prey interactions may vary depending on how strictly different species rely on visibility for their performance; brownification could in fact benefit species that are less negatively affected by such changes in visibility conditions.

The pikeperch (*Sander lucioperca*) is a common and naturally occurring piscivore in European freshwaters and is often the dominant piscivore species in turbid or brown lakes [5], [29]. Pikeperch is commonly introduced to lakes for biological control of cyprinids and for commercial and game fisheries due to its high economic value. Pikeperch introduction success [30], [31], densities [5] and growth [29] correlate positively with high water colour or turbidity. Pikeperch is an actively searching piscivore [32] that forages in open water at low light intensities [33], [34], and it could become an increasingly important piscivore with increasing brownification of lakes. In order to evaluate the potential effects of increasing brownification and pikeperch abundance on processes and patterns in freshwater lake ecosystems, it is essential to understand pikeperch predatory behaviour and potential effects on prey fish populations. If the effect of pikeperch on different prey species change with increasing brownification, and the trophic roles of the prey species differ, we should expect altered trophic and ecosystem functions as a result of changes in fish community composition.

Here, we study prey selectivity in pikeperch when foraging on European perch (*Perca fluviatilis*) and roach (*Rutilus rutilus*) in waters with different levels of humic content and during daylight and night conditions. The studies were performed in both the field and laboratory. Further, to approach the mechanisms behind patterns in prey selectivity, we studied the behaviour of pikeperch and prey fish during the different stages of the foraging cycle in laboratory experiments.

## Methods

### Field study

The field study was performed in October 2009. One clear water lake (Lake Västersjön) and one brown water lake (Lake Osbysjön), both located in Skåne, Southern Sweden, were selected for this study. Each lake was sampled with gillnets (standardized multi-mesh bottom gillnets [35]) during day and night on four occasions, resulting in eight fishing occasions for each lake, to obtain catch per unit effort (CPUE) estimates of fish compositions as a result of diel activity patterns in fish. Four multi-mesh nets were used on each sampling occasion, along with two additional nets with a mesh size of 45 mm to select for pikeperch. Nets were placed in the profundal zone at a depth of 3–9 m, the main feeding habitat of pikeperch. Each sampling occasion lasted for eight hours, from 10 to 18 o’clock during day and 22 to 06 during night samplings.

Total length of perch and roach individuals was measured (nearest mm) and the total mass per species and net was used to estimate catch per unit effort. Gut contents from all pikeperch were analysed in the lab and consumed prey were counted and identified to species. Pikeperch selectivity for roach and perch were estimated using Ivlev’s selectivity index [36].

Temperature and secchi-depth (Lake Västersjön, 9.6±0.1°C, 3.27±0.02 m and Lake Osbysjön, 10.2±0.4°C, 0.58±0.01 m, mean±SD) were measured each day of fishing. Absorbance of DOC in lake waters was measured with a spectrometer (Beckman DU 800; Beckman, Fullerton, California, USA) at 420 nm after the water was filtered through a GF/F filter (absorbance Lake Västersjön = 0.032, and Lake Osby sjön = 0.082).

### Laboratory experiments

#### Collection and maintenance of experimental fish

Six pikeperch (total length 289–341 mm, total weight 198–324 g) were caught in Lake Ringsjön, nearby Lund, Southern Sweden. Pikeperch were acclimatized to experimental conditions (16.5±0.5°C, mean±SD, 9∶15 h light:dark regime) in 500 l aquaria for five months. Four weeks before the start of the experiment pikeperch were moved and held individually in separate compartments (50×50×50 cm) of larger aquaria.

Perch were caught in Lake Hjärtasjön and Lake Ringsjön and roach were caught in Lake Ringsjön; both species were caught with dip nets. Prey fish were of similar total length (perch: 64±0.3 mm, roach: 64±0.4 mm, mean±SE), body depth (perch: 12.7±0.1 mm, roach: 12.2±0.1 mm) and wet weight (perch: 2.1±0.04 g, roach: 2.1±0.05 g). Perch and roach were acclimatized to indoor conditions for a minimum of one week in two 300 L aquaria before used in experiments. Perch were fed frozen and thawed chironomid larvae and roach were fed dry pellets every second day to maintain their condition. Pikeperch were fed live perch and roach before and between trials. To standardise feeding motivation, all pikeperch were starved for 48 h before trials. The study complies with the current laws in Sweden, no specific permits were needed for the field studies. Ethical concerns on care and use of experimental animals were followed under the permission approved for this study (M165-07) from the Malmö/Lund Ethical Committee.

#### Experimental arena and water transparency

All experiments were carried out in a transparent PVC arena (200×50×50 cm) with a water depth of 35 cm. All corners were rounded with PVC sheets to prevent prey fish from hiding or being cornered. The water temperature in the arena was kept at 16–16.5°C in all experiments. Humic water was collected from the ‘Black Pond’ (secchi depth 4 cm, pH 6.5–7.0) nearby Lund, and was used to brown colour the experimental water to the desired visual ranges. Visual range, used as a parameter for water clarity, was measured by observing a vertically held black and white secchi disk (Ø: 0.1 m) from one transparent end of the arena, and was set to the horizontal distance at which the human eye could no longer discern contrast between the black and the white disc parts [37], [38]. Humic pond water was diluted to the visual ranges 0.25, 0.5 and 2 m to be used in experiments. Absorbance of DOC was measured in the same way as the lake water (absorbance at the different visual ranges were: 0.25 m = 0.319, 0.50 m = 0.081 and 2 m = 0.022). To simulate daylight condition we provided light from two halogen spotlights (500 w), resulting in a light intensity of 500–600 lux at five mm above the water surface. Night treatments were performed in darkness (<0.001 lux).

### Prey selectivity study

Pikeperch selectivity for roach and perch was studied in the laboratory at the three visual ranges at day and night light conditions. Five individuals each of perch and roach were acclimatized in the arena for 15 minutes before a pikeperch was introduced and the experiment was started. Each trial was terminated after 45 minutes and remaining prey were counted. Each treatment combination was replicated five times, where five pikeperch participated in all treatments in a random order using a randomized block design. Individual prey fish were only used once.

Pikeperch prey preference was calculated using the Manly-Chesson selectivity index [39], ranging from 1, indicating positive selection, to 0, indicating negative selection or avoidance. In our case with two prey categories, no selectivity is indicated by an index not significantly different from 0.5.

### Behavioural study

Pikeperch and prey fish behaviours were studied in separate trials. We were neither able to observe behaviours during night conditions, nor in the 0.25 m visual range treatment, why the behavioural study was performed only in daylight conditions and in the treatments with visual ranges of 0.5 and 2 m. Five individuals each of perch and roach were acclimatized for 15 minutes in the experimental arena before one pikeperch was introduced and the trial began. Each trial was terminated after a successful attack by the pikeperch. Number of interests (when pikeperch observed a prey item) and attacks for each species were observed and counted during the trials. Observations were made behind a tarpaulin to minimize disturbances. All trials were also video recorded from the long side of the arena for further analysis of the first attack distance of pikeperch and the first escape distance for each prey species (perch and roach). Each treatment was replicated six times using six individual pikeperch in a randomized block design as above.

### Statistics

The effects of the factors visual range (random) and day/night light conditions (fixed) on pikeperch selectivity between perch and roach prey in the laboratory experiment were evaluated in a mixed effect randomized block (rb) ANOVA in SPSS (release 19). As the Manly-Chesson index for one prey species in one trial is always one minus the index for the other species, we only included the selectivity index for perch as a dependent variable. Further, as each pikeperch participated in all treatment combinations, the experimental design was not fully replicated. Including pikeperch individual identities as a blocking factor (random) in the statistical model (but not its interaction with other factors) allows for evaluation of treatment effects compensating for potential differences in levels between pikeperch individuals, as well as adjusting the degrees of freedom according to the recurring use of individuals (Quinn & Keough, 2002). The Manly-Chesson indices were further evaluated for differences from no selectivity in one-sample t-tests with the null hypothesis of an index of 0.5, again using perch indices as the dependent variable. When all prey of one species is consumed the Manly-Chesson index cannot be used. In one trial (visual range 2 m and night) all the roach were consumed, and, hence, this trial was not included in the analysis. Pikeperch and prey behavioural attributes were analysed in mixed effect rb ANOVAs, as above, for the dependent variables number of interests in prey, number of strikes, capture success, attack distance, and prey escape distance. Model residuals were not different from normal distributions (Kolmogorov-Smirnov tests, z = 0.536–1.281, p = 0.075–0.909).

## Results

### Field study

**Figure 1:**
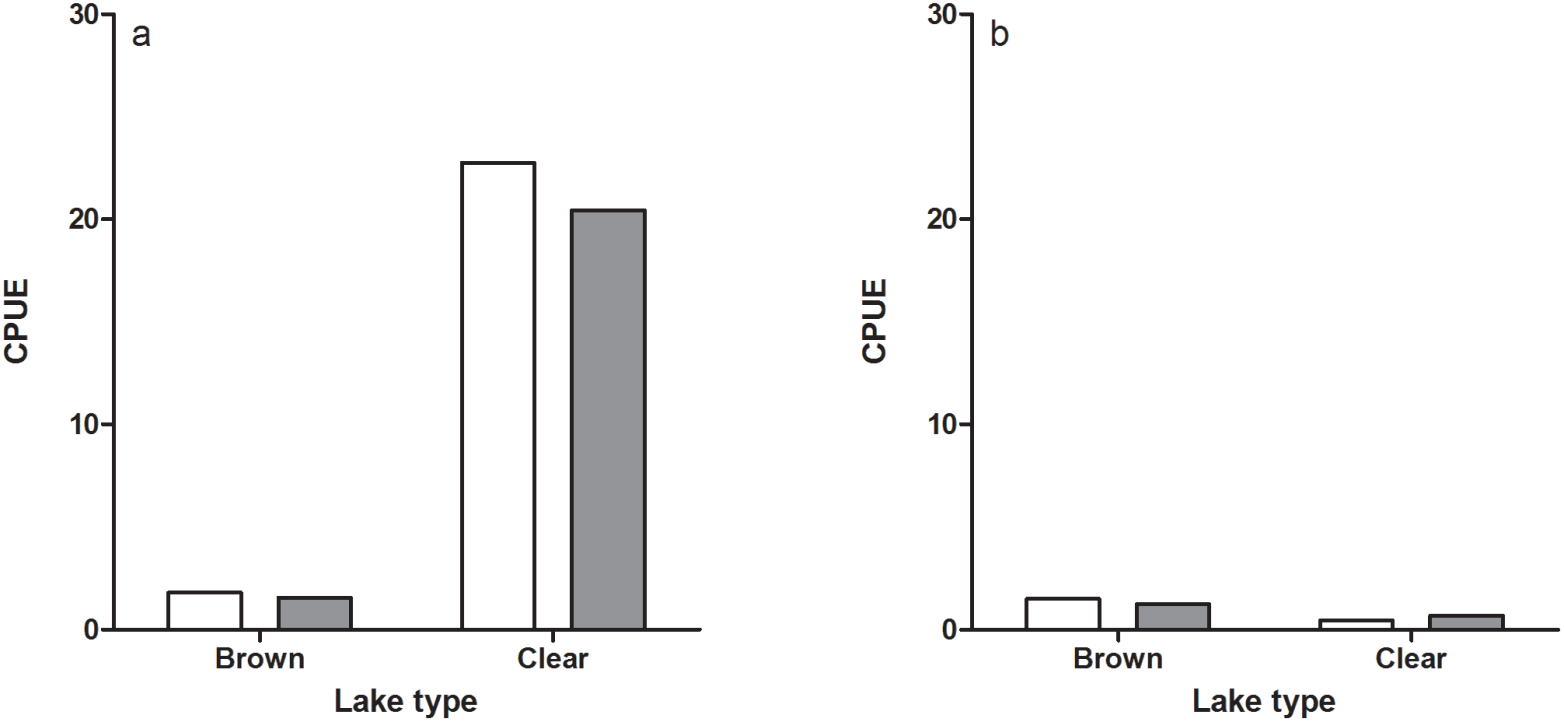
Catch per unit effort (CPUE) of perch (white bars) and roach (grey bars) in one brown and one clear water lake during day (a) and night (b).

**Figure 2:**
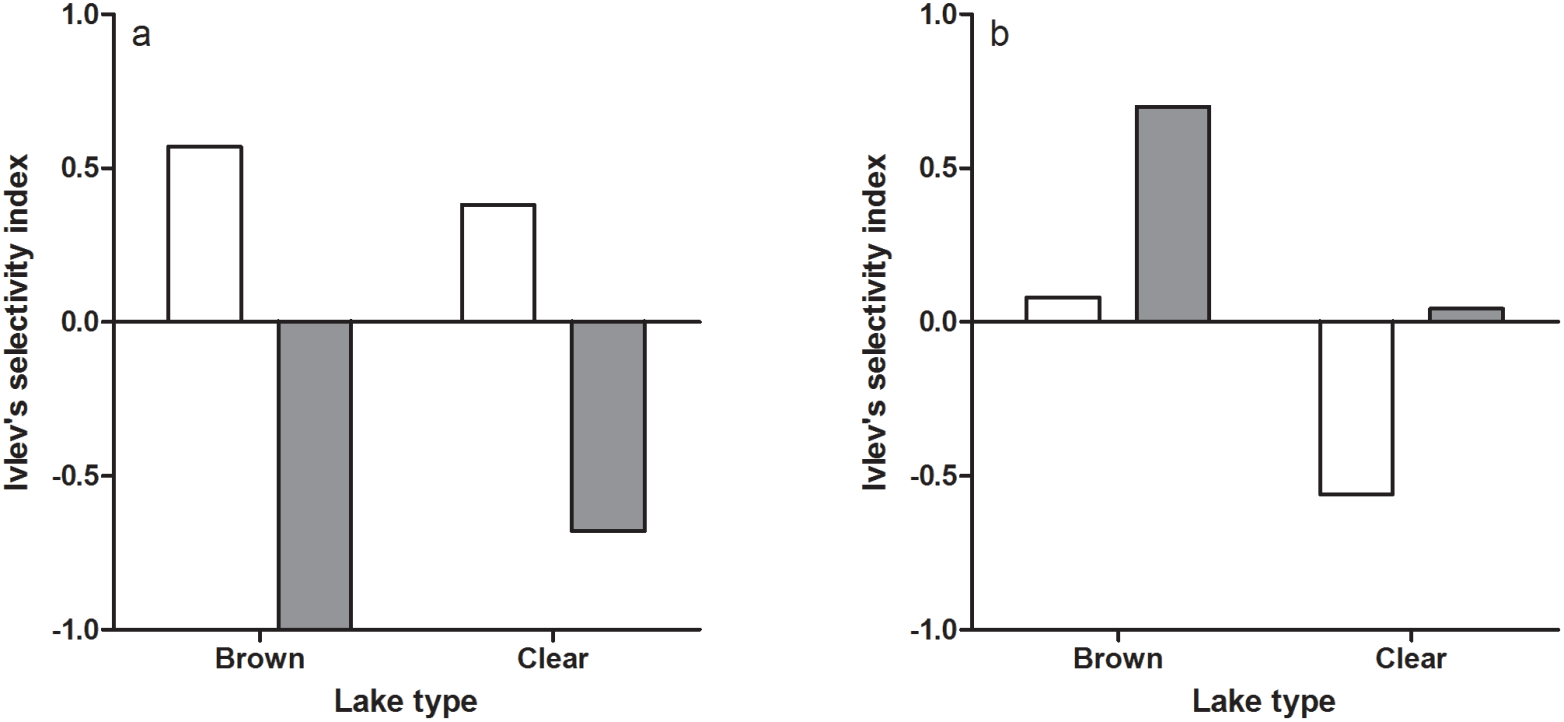
Selectivity of perch (white bars) and roach (grey bars) in one brown and one clear water lake during day (a) and night (b). The horizontal line at 0 correspond to the null hypothesis of equal selection of prey, values close to 1 represent high prey selection and values close to −1 represent low selection.

Net fishing in the clear and the brown water lake showed that there were similar densities (CPUE) of roach and perch in the two lakes (Fig. 1). However, CPUE of both species was higher during the day in the clear lake, suggesting high activity of both species during the day in this lake. The gut content analyses revealed that pikeperch showed a positive selection for perch but not of roach during the day in both lakes, as indicated by Ivlev’s selectivity index (Fig. 2a). This pattern changed at night when pikeperch selectively fed on roach in the brown water lake, whereas in the clear water lake there was no selection for roach and a negative selectivity for perch (Fig. 2b).

### Prey selectivity experiment

**Figure 3:**
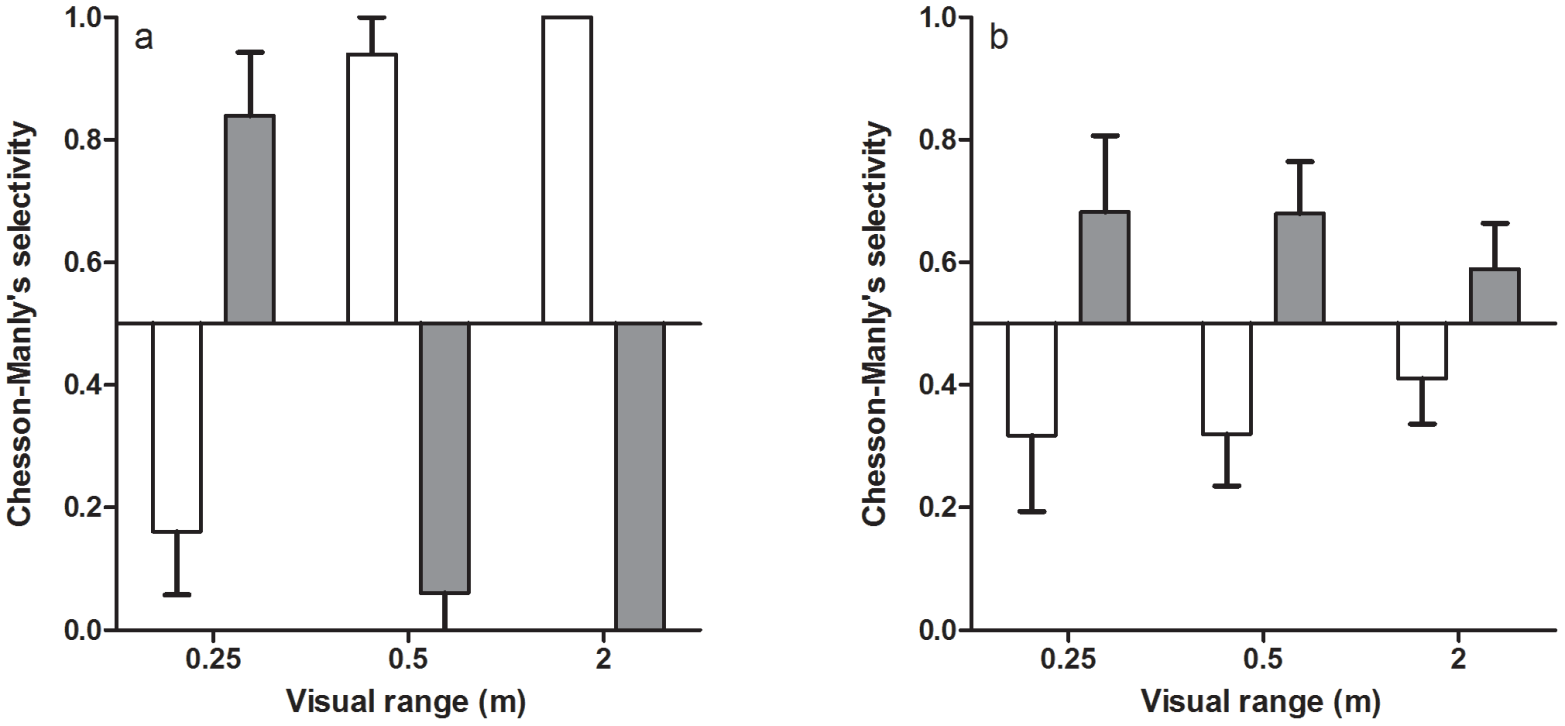
Pikeperch prey selection of perch (white bars) and roach (grey bars) at different visual ranges at day (a) and night (b) in the laboratory experiments. The horizontal line at 0.5 represents the null hypothesis of equal prey selection. Error bars denote 1 SE.

In the laboratory experiment where we studied pikeperch prey selection for roach or perch we found that there were no main effects of visual range (F_2,2_ = 1.265, p = 0.442) or light conditions (F_1,2.001_ = 1.863, p = 0.305) on the selectivity of pikeperch and, further, there was no difference among pikeperch individuals in selectivity (F_4,19_ = 0.770, p = 0.558). However, there was a significant interaction between visual range and light conditions (F_2,19_ = 12.394, p<0.001), indicating that pikeperch change their selectivity between day and night conditions and, further, that visual range in daylight conditions affect prey species selectivity (Fig. 3). One-sample t-tests for each combination of visual range and light conditions revealed that pikeperch showed a significant selection for roach at the 0.25 m visual range in daylight conditions, but selected for perch at longer visual ranges (see above; Fig. 3a). During night, pikeperch did not show any significant selection for any prey in any of the visual ranges (Table 1, Fig. 3b).

**Table 1:** The effects of light condition and visual range on prey selectivity of pikeperch.

Light conditions	Visual range (m)	df	t	p
Day	0.25	4	−3.288	0.030
Day	0.5	4	7.223	0.002
Day	2	-	-	-
Night	0.25	4	−1.473	0.215
Night	0.5	4	−2.112	0.102
Night	2	3	0.816	0.474

The treatment of daylight conditions at 2 m visibility was excluded from the analysis as all perch and no roach were eaten, resulting in no variance in data.

### Pikeperch and prey behaviours

**Figure 4:**
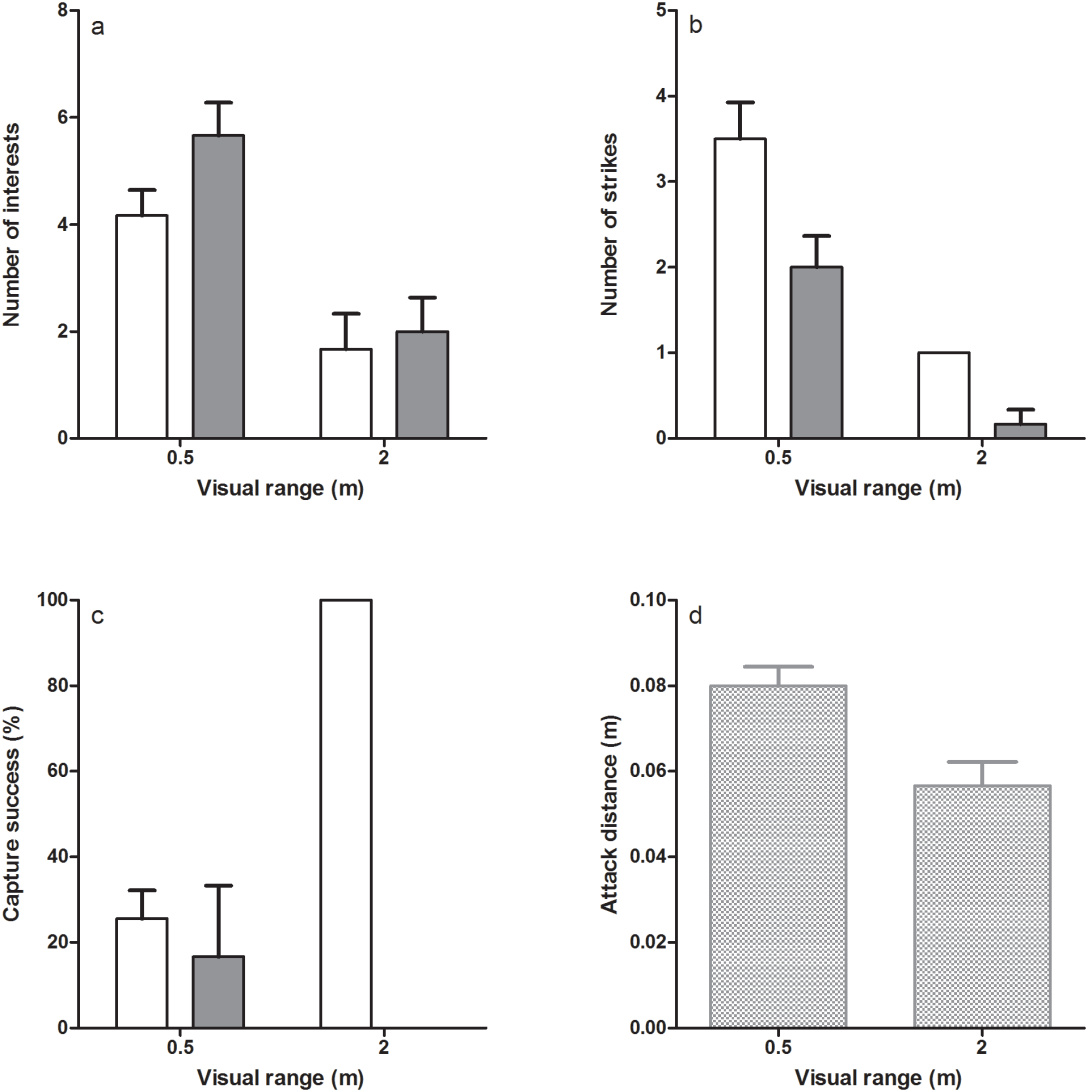
Behavioural parameters of pikeperch foraging on perch (white bars) and roach (grey bars) at visual ranges of 0.5 and 2 m. Behavioural parameters include number of interests (a) and strikes (b), as well as capture success (c). In the analyses of attack distances (d), data for the two prey species are pooled. Error bar denote 1 SE.

**Figure 5:**
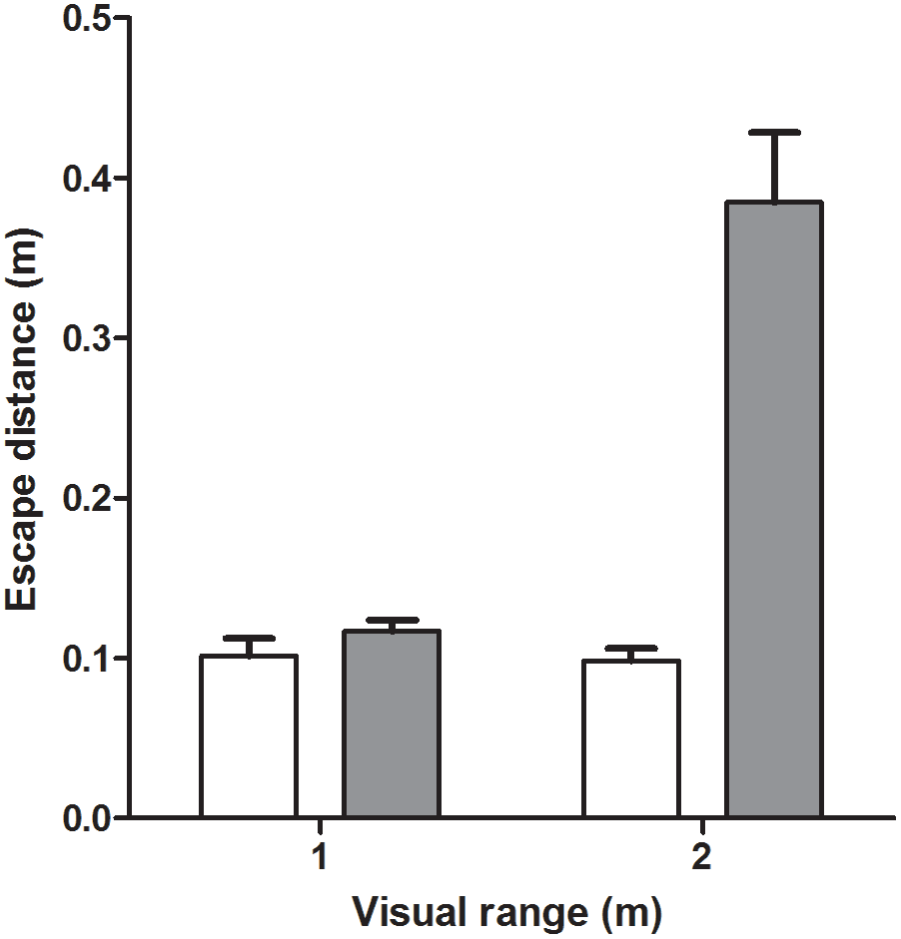
Escape distance of perch (white bars) and roach (grey bars) at a visual range of 0.5 and 2 m. Error bar denote 1 SE.

The rbANOVA revealed a significant interaction between visual range and prey species on both pikeperch capture success and prey escape distance (Table 2). With regards to capture success, the interaction effect originated from a change from a similar capture success on roach and perch prey in water with a visual range of 0.5 m to 100% capture success on perch prey and a 0% capture success on roach at a visual range of 2 m (Fig. 4c). There was no difference between species in prey escape distances in water with 0.5 m visual range, whereas escape distance in roach was substantially longer in water with 2 m visual range (Fig. 5). Pikeperch attack distance on perch prey was significantly affected by visual range (Table 2) with a longer attack distance in water with 0.5 m visual range (Fig. 4d). All other interaction terms as well as number of interests (Fig. 4a) and number of strikes (Fig. 4b), including pikeperch individual as a blocking factor, had no significant effects on the measured behaviours (Table 2).

**Table 2:** The effects of visual range (VR, random factor) and prey species (Prey, roach or perch, fixed) on pikeperch foraging behaviours.

		Number of interests		Number of strikes			Capture success			Attack distance			Escape distance		
	df	F	p	df	F	p	df	F	p	df	F	p	df	F	p
VR	1,1	27.939	0.119	1,1	42.250	0.097	1,1	0.402	0.640	**1,5**	**14.412**	**0.013**	1,1	0.952	0.508
Prey	1,1	2.469	0.361	1,1	12.250	0.177	1,1	1.428	0.444		N/A		1,1	1.233	0.467
VR*Prey	1,15	1.219	0.287	1,15	0.2153	0.281	**1,15**	**21.884**	**<0.001**		N/A		**1,15**	**32.896**	**<0.001**
PI	5,15	2.194	0.109	5,15	0.875	0.521	5,15	0.401	0.840	5,5	1.706	0.286	5,15	0.747	0.601

Pikeperch individual (PI) was included as a random blocking factor.

## Discussion

In the field study as well as in the laboratory experiments we found changes in pikeperch prey selectivity between perch and roach as the optical conditions changed, including changes in both light intensity (night/day) and brown coloration. Many studies indicate that degraded optical conditions decrease the possibilities for the predator to choose among prey resulting in absence of selectivity for any prey [11], [40], [41], whereas our results instead show a change in selectivity from one prey species to another. The field observations show selectivity for perch in daylight conditions in both brown and clear water, but at night this pattern changed and we found that there was selectivity for roach in brown water. The results from our laboratory experiments on pikeperch selectivity provide us with further details on how light and visibility conditions affect prey selectivity in pikeperch. The significant interaction between visual range and light condition in the rbANOVA along with t-tests on prey selectivity highlights two interesting aspects. It corroborates a difference in prey selectivity between day and night conditions, with no significant selectivity among perch and roach prey during night, whereas during day there was a significant prey selectivity. Moreover, the selectivity during day was dependent on the visual range as pikeperch showed a selection for roach in very poor visibility conditions (visual range = 0.25 m), whereas with increasing visual ranges there was a change in selectivity towards a preference for perch. This suggests that there may be a threshold level of degrading visual conditions, where pikeperch selectivity change from a selection for roach to a selection for perch. Such a threshold level of humic contents are to be found in lakes today [42] and may very well be reached in an increasing number of lakes if the documented brownification continues to increase according to predictions. In order to understand and predict the consequences of such potential thresholds for fish community structure it is crucial to increase our understanding of the mechanisms behind prey selectivity in pikeperch. As selectivity can be a function of active preference in the predator, differential encounter rates between prey types or differences in both predator and prey behaviour [43], [44], we used controlled behavioural experiments to evaluate possible mechanisms affecting pikeperch prey selectivity.

An active prey choice in pikeperch should be indicated by different aptitudes for attack between prey species. As neither pikeperch number of interest, nor number of attacks differed between prey species, the behavioural results suggest that active choice is not a major contributor to pikeperch selectivity. Instead, prey selectivity in pikeperch seems to be a result of processes at later stages in the foraging cycle, i.e. at the capture stage, where success can be affected by characteristics of both the predator and the prey in combination with environmental factors. We found that capture success was affected both by prey species attacked and visual range in the water. In the laboratory experiment we found a 100% capture success for pikeperch foraging on perch in clear water and a 0% capture success when foraging on roach, which thus explains why pikeperch show selectivity for perch in daylight conditions and at long visual ranges. Pikeperch attack distances were always shorter than the measured escape distances of roach in clear water, i.e. roach avoid predation by initiating an early escape response at distances that are outside the distance where pikeperch initiate their attacks, a so called safe distance also used by other fish species [45].

At the shorter visual range of 0.5 m, both capture success and escape distances are comparable between prey species. Still, results from both the lakes and the experiments show selectivity for perch at this visual range during day. Roach are known to school tightly as a response to predation threat and rely on a high swimming capacity for predator evasion, whereas perch reduce predation by fine-tuned manoeuvrability, cryptic coloration and spiny rayed fins [46]–[48]. Perch also commonly adopt an inactivity strategy when facing predation risk, which we also observed in the behavioural experiment. Schooling acts as to reduce encounter rate [49], [50] and to dilute individual risks and confuse predators [51]. These behaviours could lie behind the maintained selectivity for perch in spite of comparable capture success and escape distance among species; we observed pikeperch to generally approach their prey slowly and attack from a short distance and inactivity should be an inappropriate measure to avoid pikeperch predation. Further, the results also suggest that schooling and high swimming capacity, as in roach, is more efficient to reduce pikeperch predation rates compared to fine-tuned manoeuvrability and spiny rayed fins [48], as in perch.

During night and in highly brown water (visual range of 0.25 m) there is a relative shift towards selectivity for roach in both the lakes and experiments, although this selectivity was not significant in experiments. However, due to logistic reasons we were not able to quantify the behavioural attributes of pikeperch and prey during night and at 0.25 m visual range why mechanistic explanations of selectivity patterns under these circumstances are more speculative. The results may, however, be interpreted as a reduction of the efficiency of anti-predatory behaviours in roach with decreasing light intensity or increasing water colour, as schooling and timing of fast-start escapes could be impaired with poor visual information. Vision is a key component in school formation [52], and reduced optical condition due to light limitations or turbid/brown water results in a split up of schools [53], [54]. Further, the visual system of pikeperch is adapted for low light intensities with a specific sensory adaptation, *tapetum lucidum* [55], [56], which should enhance the ability of pikeperch to detect prey in brown water environments. Differences in visual capacity in pikeperch and roach during poor light conditions may affect the relative disadvantage due to differences in escape and detection distances present in daylight conditions. Further, feeding efficiency of pikeperch has been shown to be unaffected by light condition and turbidity [57], which indicates that pikeperch may use sensory input from the lateral line system when foraging in poor optical conditions [58]. Thus, a combination of reduced efficiency of behavioural responses in prey and a relative advantage for the predator due to better vision/sensory input in poor optical conditions may facilitate the change in selectivity found in pikeperch at night and in very brown waters.

Pikeperch are well adapted for foraging under visually degraded conditions compared with other sympatric piscivores [57], [59]. This suggests that pikeperch should be an increasingly important piscivore in a future, browner lake scenario. As piscivore predation rates and prey selectivity can impose far-reaching structuring forces on fish communities and trophic processes, environmentally driven prey selectivity in pikeperch holds important cues to our understanding of lake system processes in a changing environment. This may be especially true for lake systems undergoing major brownification processes, as they are commonly signified by relatively low productivity [24], where selective predation should assert a strong structuring force on lower trophic levels. Our work highlights the importance of considering piscivore-prey interactions and visibility conditions in evaluations and predictions of trophic effects from the increasing brownification of lake ecosystems.

## Supporting Information

Data S1 **Data behind** **fig. 1** **–** **5** **.**
(XLSX)
